# Spanish *HTT* gene study reveals haplotype and allelic diversity with possible implications for germline expansion dynamics in Huntington disease

**DOI:** 10.1093/hmg/ddac224

**Published:** 2022-09-20

**Authors:** Ainara Ruiz de Sabando, Edurne Urrutia Lafuente, Arkaitz Galbete, Marc Ciosi, Fermín García Amigot, Virginia García Solaesa, Spanish HD Collaborative group, Victoria Álvarez Martínez, Asunción Martinez-Descals, Montserrat Mila, Maria José Trujillo-Tiebas, Jose Luis López-Sendón, María Fenollar-Cortés, Inés Legarda, Sara Bernal Noguera, Jose M Millán, Camen Durán-Herrera, Darren G Monckton, Maria A Ramos-Arroyo

**Affiliations:** Department of Medical Genetics, Hospital Universitario de Navarra, IdiSNA, Pamplona 31008, Spain; Department of Health Sciences, Universidad Pública de Navarra, IdiSNA, Pamplona 31008, Spain; Fundación Miguel Servet-Navarrabiomed, IdiSNA, Pamplona 31008, Spain; Fundación Miguel Servet-Navarrabiomed, IdiSNA, Pamplona 31008, Spain; Department of Statistics, Informatics and Mathematics, Universidad Pública de Navarra, IdiSNA, Pamplona 31006, Spain; School of Molecular Biosciences, College of Medical, Veterinary and Life Sciences, University of Glasgow, Glasgow G12 8QQ, UK; Department of Medical Genetics, Hospital Universitario de Navarra, IdiSNA, Pamplona 31008, Spain; Department of Medical Genetics, Hospital Universitario de Navarra, IdiSNA, Pamplona 31008, Spain; School of Molecular Biosciences, College of Medical, Veterinary and Life Sciences, University of Glasgow, Glasgow G12 8QQ, UK; Department of Medical Genetics, Hospital Universitario de Navarra, IdiSNA, Pamplona 31008, Spain; Fundación Miguel Servet-Navarrabiomed, IdiSNA, Pamplona 31008, Spain

## Abstract

We aimed to determine the genetic diversity and molecular characteristics of the Huntington disease (HD) gene (*HTT*) in Spain. We performed an extended haplotype and exon one deep sequencing analysis of the *HTT* gene in a nationwide cohort of population-based controls (*n* = 520) and families with symptomatic individuals referred for HD genetic testing. This group included 331 HD cases and 140 carriers of intermediate alleles. Clinical and family history data were obtained when available. Spanish normal alleles are enriched in C haplotypes (40.1%), whereas A1 (39.8%) and A2 (31.6%) prevail among intermediate and expanded alleles, respectively. Alleles ≥ 50 CAG repeats are primarily associated with haplotypes A2 (38.9%) and C (32%), which are also present in 50% and 21.4%, respectively, of HD families with large intergenerational expansions. Non-canonical variants of exon one sequence are less frequent, but much more diverse, in alleles of ≥27 CAG repeats. The deletion of CAACAG, one of the six rare variants not observed among smaller normal alleles, is associated with haplotype C and appears to correlate with larger intergenerational expansions and early onset of symptoms. Spanish HD haplotypes are characterized by a high genetic diversity, potentially admixed with other non-Caucasian populations, with a higher representation of A2 and C haplotypes than most European populations. Differences in haplotype distributions across the CAG length range support differential germline expansion dynamics, with A2 and C showing the largest intergenerational expansions. This haplotype-dependent germline instability may be driven by specific *cis*-elements, such as the CAACAG deletion.

## Introduction

Huntington disease (HD; OMIM 143 100) is an autosomal dominant neurodegenerative disorder caused by a pathogenic expansion of the CAG trinucleotide in the huntingtin (*HTT*) gene. Alleles between 36 and 39 CAGs present with reduced penetrance, whereas those of ≥40 repeats are fully penetrant (1). Below 36 CAGs, alleles are not considered disease-causing; however, alleles of 27–35 CAGs, known as intermediate alleles (IAs), can potentially undergo germline expansions into the HD range (2).

HD occurs in different populations across the globe, but it presents notable geographical differences, with higher prevalence in Europe and its descendant populations, in contrast to the lower prevalence observed in Asian and African populations (3). Various studies have explored the origins of HD chromosomes through the analysis of single nucleotide polymorphisms (SNPs) and other polymorphisms around the *HTT* gene, attributing differences in population prevalence to their *HTT* haplotype makeup (4). Warby *et al*. (5) showed that two major haplotypes, A1 and A2, are strongly associated with HD chromosomes in Caucasians. These two major haplotypes are, however, absent in Asian and African HD chromosomes, where C, and other A and B haplotypes seem to be more prevalent (6–9). They speculated that differences in the *HTT* haplotypes among HD populations may indicate the existence of *cis*-elements conferring different expansion mechanisms to the CAG tract. Other authors have suggested that A haplotypes are likely to act as a reservoir of new HD mutations because high normal and IAs might be more likely to expand into the HD range (10).

A new research line on *cis*-CAG tract elements of the *HTT* gene has focused on the DNA sequence structure of exon one, and more specifically, on the sequence immediately downstream of the CAG repeats. In a typical allele, the glutamine-encoding polymorphic CAG sequence is followed immediately downstream by the glutamine-encoding CAACAG. Next downstream, is the proline-encoding CCGCCA, which is followed by the proline-encoding polymorphic CCG repeat sequence and by CCTCCT. Variations in the number of glutamine-encoding CAA have been associated with modifications in age at onset (11–13), possibly because of different protective or pathogenic mechanisms. Some variants are associated with a delayed age of HD motor onset, whereas others with an earlier presentation because of an increase in somatic instability of the CAG tract relative to that predicted by the length of the entire polyglutamine-encoding array.

We present the first molecular study of the *HTT* gene region in the Spanish population across the normal, intermediate and expanded CAG alleles through a detailed extended haplotype analysis that includes sequencing of exon one. The results unmask a great genetic heterogeneity that allows a comparative evaluation of potential haplotype-dependent germline dynamics, most likely under the effect of specific *cis*-elements.

## Results

### CAG length distribution

We estimated the size of the *HTT* CAG repeat of 991 independent individuals: 331 HD subjects, 140 IAs carriers and 520 population-based controls. The results are shown in Table 1.

**Table 1:** Description of *HTT* gene alleles among index members of the study cohorts: population-based, intermediate allele (IA) carriers and Huntington disease (HD) subjects

	Population-based cohorts	IA carriers		HD cases	
		Small allele	Large allele	Small allele	Large allele
Number of alleles	1040	140	140	331	331
CAG length range	8–34	10–30	27–35	8–37	36–70
Most common CAG size (%)	17 (30.3)	17 (35)	28 (25)	17 (26.3)	42 (17.2)
Mean CAG (SD)	18.4 (3.3)	18.4 (3.3)	29.4 (2.3)	18 (3.7)	43.6 (4.8)
% High-normal alleles (20–26 CAGs)	23.9 *n* = 249	25.7 *n* = 36	—	19 *n* = 63	—
% IAs (27–35 CAGs)	2.5 *n* = 26	2.1 *n* = 3	100	3 *n* = 10	—
% HD alleles (>35 CAGs)	—	—	—	0.6 *n* = 2	100

*Note:* Population-based cohorts versus (i) small allele of IA carriers, *P* = 0.973, and (ii) small allele of HD cases, *P* = 0.079.

In the general population, the most frequent allele contained 17 CAGs, with a mean length of 18.4 CAG repeats (standard deviation, SD = 3.3), and a frequency of high-normal alleles and IAs of 23.9 and 2.5%, respectively. This CAG distribution was not significantly different from those of the small alleles of IA carriers or HD subjects (*P* = 0.973 and *P* = 0.079, respectively). Small alleles of IA carriers showed very similar mean CAG length (18.4; SD = 3.3) and frequencies of high-normal (25.7%) and intermediate (2.1%) alleles. Small alleles of HD subjects, however, presented lower mean CAG length (18; SD = 3.7), lower frequency of high-normal chromosomes (19%), higher frequency of IAs (3%) and the presence of two HD reduced-penetrance alleles (0.6%), with 36 and 37 CAGs.

### Haplotypes

**Figure 1:**
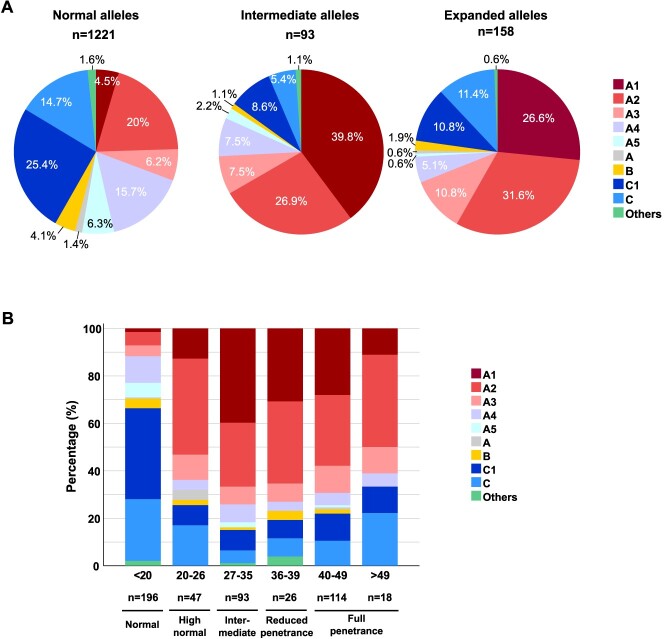
*HTT* haplotype distribution across the CAG range in alleles of the Spanish population represented in (**A**) three categories (normal, intermediate and expanded alleles), and (**B**) six categories, subclassing the normal and expanded alleles.

Genotype information was obtained for a total of 1401 individuals. Haplotypes were CAG-phased and index family members selected, resulting in a total of 1472 independent chromosomes: 1221 normal (243 phased), 93 intermediate and 158 expanded (Fig. 1A).

Haplotypes C1 and C were most prevalent among normal chromosomes (40.1%), comprising 14% of IAs and 22.2% of HD alleles. Haplotypes A1, A2 and A3 were significantly enriched in IAs and HD alleles (74.2% and 69%, respectively), compared with normal alleles (30.7%). A2 was the predominant haplotype among expanded chromosomes (31.6%), showing slightly decreasing frequencies among IAs (26.9%) and normal alleles (20%). Haplotype A1, however, was present in 26.6% of expanded alleles and was the most frequent haplotype among IAs (39.8%), but it only represented 4.5% of normal alleles.

To further analyze the haplotype makeup of the Spanish population, the three main allele categories (normal, intermediate and expanded) were split into smaller CAG ranges. As shown in Figure 1B, haplotype distribution changes drastically from the 20 CAG allele onwards. C haplotypes were highly frequent (64.3%) in smaller alleles, with lesser representation among longer allele chromosomes, primarily in those with 20–49 CAGs (19.3%). On the contrary, A1, A2 and A3 only represented 11.7% of < 20 CAG alleles, but over 60% of all other allele groups: high-normal, intermediate and expanded. A1 peaked among IAs (39.8%), losing representation as the number of CAG increased: 30.8% in reduced-penetrance alleles, 28.1% in 40–49 CAG alleles and 11.1% in > 49 CAG alleles. With respect to haplotype A2, it was most frequent among high-normal alleles (40.4%), less so between alleles of 27–49 CAGs (29.2%) though highly prevalent in alleles of 50 CAGs or more (38.9%).

In order to assess the possible influence of HD haplotypes on the CAG repeat length, we analyzed the CAG distributions of the small allele of IA carriers and HD cases, representing the general population (*n* = 252), and of expanded alleles (*n*=158) (Table 2). The CAG distribution of the small allele cohort showed haplotype-dependent differences (analysis of variance, ANOVA *P* < 0.001), with a higher mean in haplotypes A1 (22.6 ± 7.1, *n* = 12) and A2 (20.4 ± 2.8, *n* = 31) compared with C1 (17.2 ± 2, *n* = 80) and C (16.6 ± 3.5, *n* = 59). For expanded alleles, in contrast, the highest CAG averages were not found in the A1 (42.1 ± 3.3, *n* = 42), A2 (43.9 ± 6.3, *n* = 50) or A3 (44.6 ± 5.5, *n* = 17), the most common HD haplotypes, but rather in the more rare A4 (45.6 ± 10.2, *n* = 8), C1 (45.1 ± 5.2, *n* = 17) and C (47.1 ± 7.6, *n* = 18) haplotypes. Moreover, the largest CAG alleles were found in A4 (70), C (68) and A2 (66, 62). CAG distribution differences by haplotypes for expanded alleles, however, did not reach statistical significance (ANOVA *P* = 0.067).

**Table 2:** CAG length distribution, by *HTT* gene haplotype, in alleles representing the general population (small allele of IA carriers and HD subjects) and expanded alleles

**Haplotype**	**Small alleles of IA carriers and HD subjects, representing the general population**		**Expanded alleles**	
	*N*	CAG average ± SD	*N*	CAG average ± SD
**A1**	12	22.6 ± 7.1	42	42.1 ± 3.3
**A2**	31	20.4 ± 2.8	50	43.9 ± 6.3
**A3**	15	19.5 ± 4.2	17	44.6 ± 5.5
**A4**	26	18.3 ± 4.5	8	45.6 ± 10.2
**A5**	12	15.8 ± 1.1	1	48
**B**	10	19.4 ± 3.2	3	41.7 ± 2.3
**C1**	80	17.2 ± 2	17	45.1 ± 5.2
**C**	59	16.6 ± 3.5	18	47.1 ± 7.6
**Others**	7		2	
**Total**	252		158	
**ANOVA**	*P* < 0.001		*P* = 0.067	

### *HTT* exon one sequence

The downstream sequence of the CAG repeat in exon one of *HTT* gene was analyzed in 280 unrelated subjects, which included 280 normal alleles, 179 IAs and 101 expanded alleles. The frequency of atypical sequences was higher among alleles < 27 CAGs (7.1%, *n* = 20) than in intermediate (4.5%, *n* = 8) or expanded alleles (5.9%, *n* = 6). Longer alleles (≥27 CAGs), however, presented a greater variety of structures. Among intermediate and expanded alleles, we identified six atypical structures, not seen among alleles of < 27 CAGs, whereas only one (an additional CCT, on haplotype C) was unique to normal alleles. Detailed sequences and frequencies of the atypical alleles are shown in Table 3.

**Table 3:** *HTT* gene exon one sequencing structures identified in 560 non-related normal, intermediate and expanded alleles

***HTT* exon one variant annotation**	**Haplotype**	**% alleles** **(***n***)**	**% alleles among CAG < 27** **(***n***)**	**% alleles among CAG 27–35** **(***n***)**	**% alleles among CAG > 35** **(***n***)**	**CAG repeat length, age at onset**
Typical structures (canonical sequences)						
(CAG)_10 to 66_(CAACAG)_1_ (CCGCCA)_1_(CCG)_6 to 12_(CCT)_2_	A, B, C	93.9% (526)	92.9% (260)	95.5% (171)	94.1% (95)	
Atypical structures (non-canonical sequences)						
(CAG)*_x_*(CAACAG)_2_(CCGCCA)_1_ (CCG)_7_(CCT)_3_ *x* = 15 [5], 16 [2], 22, 23 [2], 29, 49 [2]	C	2.3% (13)	3.6% (10)	0.6% (1)	2% (2)	49 CAGs, 41 years 49 CAGs, 37 years
(CAG)*_x_*(CAACAG)_1_(CCGCCA)_0_ (CCG)_9_(CCT)_2_ *x* = 18 [4], 19 [2], 39	B	1.3% (7)	2.1% (6)	0	1% (1)	39 CAGs, 68 years
(CAG)*_x_*(CAACAG)_1_(CCGCCA)_1_ (CCG)_9_(CCT)_3_ *x* = 16, 18 [3]	C	0.7% (4)	1.4% (4)	0	0	
(CAG)*_x_*(CAA)_1_(CAACAG)_1_ (CCGCCA)_1_(CCG)_7_(CCT)_2_ *x* = 30, 33, 38	A1, A2	0.5% (3)	0	1.1% (2)	1% (1)	38 CAGs, 74 years
(CAG)_19_(CAA)_1_(CAG)_10_ (CAACAG)_1_(CCGCCA)_1_(CCG)_7_ (CCT)_2_	A2	0.4% (2)	0	1.1% (2)	0	
(CAG)*_x_*(CAACAG)_1_(CCGCCA)_0_ (CCG)_12_(CCT)_2_ *x* = 30	C1	0.2% (1)	0	0.6% (1)	0	
(CAG)*_x_*(CAACAG)_0_(CCGCCA)_1_ (CCG)_7_(CCT)_2_ *x* = 30	A2	0.2% (1)	0	0.6% (1)	0	
(CAG)*_x_*(CAACAG)_0_(CCGCCA)_1_ (CCG)_10_(CCT)_2_ *x* = 38	C	0.2% (1)	0	0	1% (1)	38 CAG, asymptomatic at 54 years 44 CAG, 31 years
(CAG)*_x_*(CAACAG)_0_(CCGCCA)_0_ (CCG)_12_(CCT)_2_ *x* = 33, 38	C1	0.4% (2)	0	0.6% (1)	1% (1)	42 CAG, 32 years 38 CAG, 63 years
Total		560	7.1% (14)	4.5% (8)	5.9% (6)	

*Note: x* = Number of CAG; [] = Number of alleles. The variant annotation is based on the *HTT* locus reference sequence NM_001388492.

The two most common atypical structures overall were the duplication of CAACAG, on haplotype C, and the deletion of CCGCCA, on haplotype B. Their frequencies were higher among normal alleles (3.6% and 2.1%, respectively) than in IAs (0.6% and 0%, respectively) and expanded alleles (2% and 1%, respectively).

**Figure 2:**
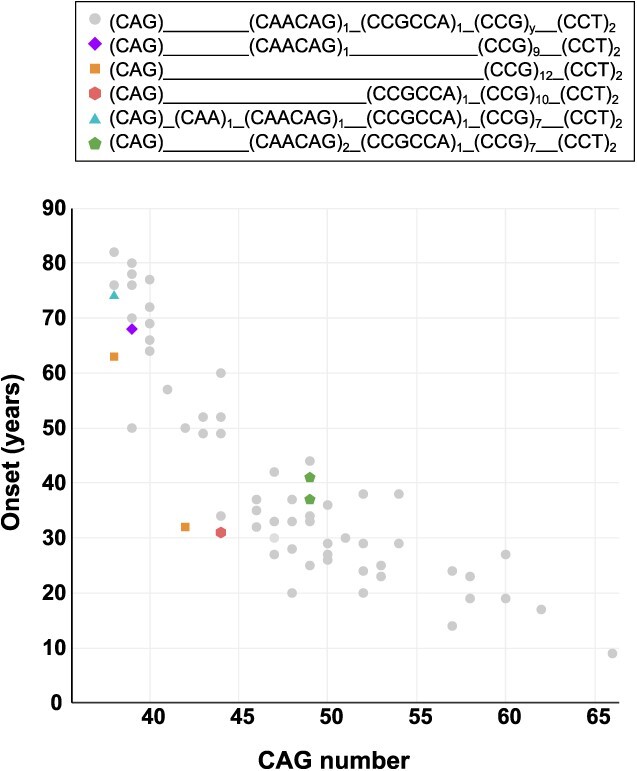
Age at onset by number of CAGs in the different exon one structures (*n* = 68).

HD cases with CAACAG duplication and 49 CAG repeats presented delayed onset of symptoms (37 and 41 years) compared with the model for prediction of age at onset, based on CAG number, by Langbehn *et al*. [33 years; (15)]. The CCGCCA deletion was identified in one patient, carrier of a 39 CAG expansion, with onset at 68 years. The age at onset by CAG length for the different exon 1 structures are represented in Figure 2.

Additional atypical structures were identified among IAs and HD cases. CAA interruptions were observed in two different structures. First, an additional CAA codon immediately after the CAG repeats, on haplotypes A1 and A2, was identified in three alleles of 30, 33 and 38 CAGs. The 38 CAG allele carrier presented with symptoms at 74 years. Second, an interruption directly in the middle of the CAG sequence (i.e. allele structure (CAG)_19_(CAA)_1_(CAG)_10_(CAACAG)_1_(CCGCCA)_1_(CCG)_7_(CCT)_2_, on haplotype A2.

The 12 nucleotide sequence between the CAG and CCG repeats presented deletions in ≥27 CAGs alleles. Two CAACAG deletions were found in alleles with 30 and 38 CAGs, on haplotypes A2 and C, respectively. The CAACAGCCGCCA deletion was identified in two alleles of 33 and 38 CAGs, both on haplotype C1. These deletion-carrying alleles appeared to be highly unstable in the male germline and anticipated the onset of HD symptoms, as we observed that: (i) the chromosome with (CAG)_38_(CAACAG)_0_(CCGCCA)_1_(CCG)_10_(CCT)_2_ expanded to a 44 CAG allele in the next generation, and the carrier’s onset of symptoms presented 14 years earlier than that predicted by the CAG number (15); (ii) a new mutation event occurred in the family with the IA (CAG)_33_(CAACAG)_0_(CCGCCA)_0_(CCG)_12_(CCT)_2_, and the resulting 42 CAG allele carrier showed symptoms 20 years earlier compared with the prediction by CAG number (15) and (iii) a subject, carrying the allele (CAG)_38_(CAACAG)_0_(CCGCCA)_0_(CCG)_12_(CCT)_2_, similarly showed symptoms 14 years earlier than the predicted age at onset by CAG number (15).

To examine if the differences in *HTT* exon 1 structure had an impact on age at onset, we classified variants by the number of CAAs and performed a linear regression model, adjusted by the number of CAGs. By comparing the age at onset of cases with CAA_1_ (*n* = 62) with those with CAA_0_ (*n* = 3) and CAA_2_ (*n* = 3), we observed that the deletion (CCA_0_) was associated with earlier onset (*P* = 0.011), showing an advanced clinical presentation of approximately 15 years (95% CI: 3.7, 26.9). On the contrary, the duplication (CCA_2_) did not show an effect in age at onset (*P* = 0.522; *b* = 3.7, 95% CI: −7.7, 15.1).

### Families with large intergenerational CAG tract length changes

**Figure 3:**
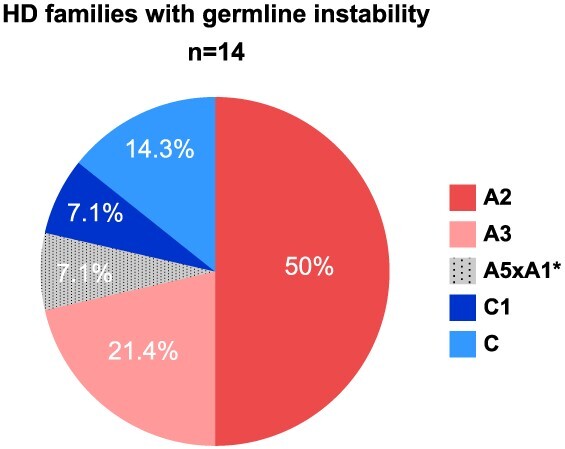
Pie chart representing the haplotype makeup of HD families with unstable CAG transmissions (>5 CAGs). ^*^A5xA1 is a recombinant haplotype, comprising portions of A5 and A1 haplotypes.

We selected 14 families with an intergenerational length change event of >5 CAGs (Table 4). Of them, six were IAs that expanded into an HD allele. Haplotype analysis showed that these large expansions occurred predominantly in haplotypes A2 (50%) and A3 (21.4%), followed by C (14.3%), C1 (7.1%) and A5xA1 (7.1%; Fig. 3). All intergenerational expansions of known parental origin were paternally transmitted (*n* = 17); there were also two contraction events, one of four CAGs, from a maternal allele, and another of one CAG, from a paternal allele. Regarding the sequence of *HTT* exon one, most germline unstable alleles had a canonical structure, with the exception of two atypical variants that carried the deletions of CAACAG and CAACAGCCGCCA. These deletions appeared more frequently in HD families with large intergenerational expansions than in the rest of the HD families (14.3% versus 1.1%; *P* = 0.044).

**Table 4:** *HTT* gene molecular characteristics of Huntington disease families with intergenerational changes of > 5 CAGs

**Family**	***HTT* exon one variant annotation**	***HTT* Haplotype**	**CAG number of intergenerational transmission**	**CAG number of expansion/ contraction events**	**Parental transmission**
FAM_1	(CAG)_33_(CAACAG)_0_(CCGCCA)_0_(CCG)_12_(CCT)_2_	C1	33–>42 33–>32	+9 −1	Paternal Paternal
FAM_2	(CAG)_32_(CAACAG)_1_(CCGCCA)_1_(CCG)_7_(CCT)_2_	A3	32^*^–>43	11^*^^*^	Unknown
FAM_3	(CAG)_32_(CAACAG)_1_(CCGCCA)_1_(CCG)_7_(CCT)_2_	A2	32–>43	+11	Paternal
FAM_4	(CAG)_33_(CAACAG)_1_(CCGCCA)_1_(CCG)_7_(CCT)_2_	A3	33^*^–>43 43–>39	10^*^^*^ −4	Unknown Maternal
FAM_5	(CAG)_33_(CAACAG)_1_(CCGCCA)_1_(CCG)_7_(CCT)_2_	A2	33–>43	+10	Paternal
FAM_6	(CAG)_34_(CAACAG)_1_(CCGCCA)_1_(CCG)_7_(CCT)_2_	A2	34–>47 47–>51 47–>70	+13 +4 +23	Paternal Paternal Paternal
FAM_7	(CAG)_36_(CAACAG)_1_(CCGCCA)_1_(CCG)_7_(CCT)_2_	A2	36–>43 36–>42	+7 +6	Paternal Paternal
FAM_8	FAIL (37 CAGs)	A3	37–>49	+12	Paternal
FAM_9	(CAG)_38_(CAACAG)_0_(CCGCCA)_1_(CCG)_10_(CCT)_2_	C	38–>44	+6	Paternal
FAM_10	(CAG)_39_(CAACAG)_1_(CCGCCA)_1_(CCG)_7_(CCT)_2_	A5xA1	39–>47	+8	Paternal
FAM_11	(CAG)_40_(CAACAG)_1_(CCGCCA)_1_(CCG)_7_(CCT)_2_	A2	40^*^–>47	7^*^^*^	Paternal
FAM_12	(CAG)_44_(CAACAG)_1_(CCGCCA)_1_(CCG)_7_(CCT)_2_	C	44^*^–>53	9^*^^*^	Paternal
FAM_13	(CAG)_44_(CAACAG)_1_(CCGCCA)_1_(CCG)_7_(CCT)_2_	A2	44–>66	+22	Paternal
FAM_14	(CAG)_46_(CAACAG)_1_(CCGCCA)_1_(CCG)_7_(CCT)_2_	A2	46–>58 46–>54 46–>47	+12 +8 +1	Paternal Paternal Paternal

^*^Smallest CAG number allele observed among siblings with the same HD-associated chromosome. ^*^^*^Estimated CAG expansion when parental CAG length was not available. The variant annotation is based on the *HTT* locus reference sequence NM_001388492.

### Bootstrap validation

Statistic results obtained by bootstrap methods (sampling with replacement) confirmed the accuracy of statistical estimates derived from the original dataset (Supplementary Material, Table S1), as they were very similar to those obtained with the traditional approach. Because bootstrapping may underestimate the variability of the true sample distribution (avoiding the participation of near-end values), the statistics derived from the original sample are shown throughout the results of the study, with their bootstrapping counterparts shown in Supplementary Material, Table S1.

## Discussion

We present a detailed molecular study of the *HTT* gene across the Spanish population, including SNP analysis and sequencing of exon one, to determine CAG repeat length and haplotype makeup in alleles spanning from normal to large expansions. We give special attention to the IA range (27 to 35 CAG repeats).

The epidemiology of HD seems to be related to the distribution of the CAG repeat length, the rate of new mutations and the associated haplotypes of the *HTT* gene in a given population (4). As expected, our study shows that the mean CAG repeat length, the frequency of high-normal alleles and the frequency of IAs in Spain, are in the range of other European countries (4), and, more so, of those of Southern Europe (16). If we compare our molecular results with data from other populations, the estimated HD Spanish prevalence is in the range of 4–9 per 100 000 (4). This figure is in concordance with results of a recent epidemiologic study, carried out in the north of Spain (4.9/100 000), using several health information systems, detailed family history and laboratory genetic data (17). Similar figures have also been observed in other northern and southern Spanish regions (18,19), confirming the relatively low HD prevalence within European countries.

The origin of the HD chromosomes and their correlation with prevalence in a given population have been explored through haplotype analysis (5,14,20). Haplotypes A1 and A2 are associated with HD alleles in populations of European ancestry (6,21), where HD prevalence is higher. Less is known about the *HTT* genetic background in populations of non-European descent. HD chromosome studies in Asia, show predominantly C and A5 haplotypes, and an absence of A1 and A2, both in control and expanded alleles (6–8). In the Middle East, two small studies point at A2 as the predominant HD haplotype (22,23), whereas the only African study, conducted in South Africa, identified C and B2 as the most prevalent haplotypes among expanded chromosomes of African descent (9). As expected, Spanish HD chromosomes are enriched in haplotype A1, A2 and A3 (~70% of all expanded alleles), with higher relative representation of A1 (6-fold) than A2 and A3 (1.5-fold) haplotypes, with respect to normal alleles. Spanish HD haplotype distribution, however, shows some noticeable differences with most north-western European and North American populations. First, A2 haplotype is more frequent than A1 haplotype. Second, haplogroup C, which is rare (0–4.5%) in HD European chromosomes (21), is present in 22% of HD chromosomes of Spanish origin, half of which corresponds to C1 variant. That is, the specific prevalence of HD on haplogroup C in our population would be estimated at 1.1 in 100 000, seven times higher than that calculated for north-western European countries (6). A similar overrepresentation of A2 and C1 variants has also been reported in a cohort of 317 HD subjects in Portugal (24) and 66 from southern Italy (22). This larger contribution of A2 and C haplotypes to HD mutations in the south of Europe supports the hypothesis of a worldwide geographical gradient of HD haplotypes, with A1 predominating in northern Europe and America, A2 in the south of Europe and the Middle East and C in Asia and Africa.

**Figure 4:**
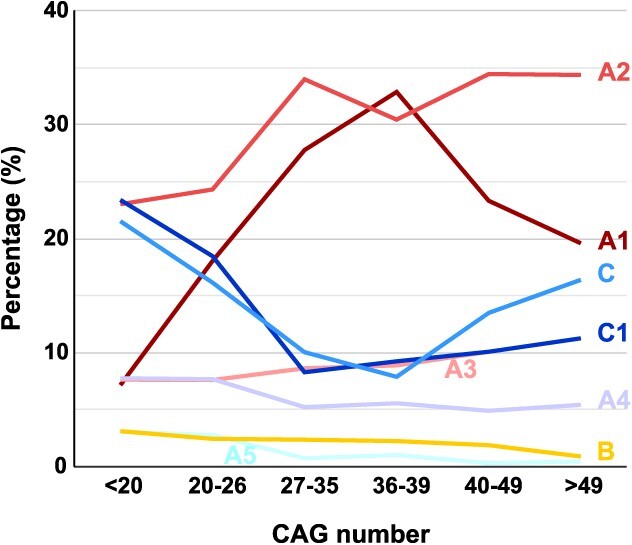
Moving averages representing haplotype frequencies of the *HTT* gene throughout the CAG range.

Analysis of haplotype distribution across different CAG range groups also reveals interesting findings. It is noticeable that different Spanish *HTT* haplotypes, other than A1, are prone to large expansions (Fig. 3). Similar observations were reported in the Portuguese population (24), arguing that, instead of a major predisposing haplotype, there was a pool of high normal alleles in each different founder haplotype more prone to give rise to pathogenic alleles. Our data, however, show that *HTT* haplotypes follow distinct trends in frequency from normal to highly expanded chromosomes, suggesting differences in their propensity to intergenerational CAG expansions. Although some HD chromosomes are likely to have originated from an IA reservoir, others seem to have the capacity to expand from smaller CAG lengths. As observed in Figure 4, haplotype A1, very uncommon in control chromosomes, is highly enriched in IAs, which most likely act as precursors of the expanded alleles. In contrast, Spanish haplogroup C chromosomes are particularly frequent in small CAG alleles and larger HD chromosomes (≥50 CAGs), but not so among IAs. Along with these findings, others and we have observed that the mean CAG repeat length for C haplotypes among HD cases is higher than for non-C haplotypes, whereas the reverse situation is observed for normal chromosomes (6). On the other hand, haplotype A2, the most frequent among HD chromosomes, and even more among larger HD chromosomes (38.9% in ≥50 CAGs), is very prevalent in high-normal alleles (40.4%), but less so in IAs (26.9%). It is, therefore, conceivable that while most expanded haplotype A1 originate from IAs, C and A2 chromosomes might be susceptible to larger expansions, giving rise to HD mutations derived from a wider range of CAG length alleles.

To better understand the different expansion patterns among Spanish alleles, we analyzed 14 HD families with high intergenerational CAG instability, showing that larger expansions occurred preferentially on haplotypes A2, A3 and C. Unstable transmissions on haplotypes A2 and A3 have been observed in Portuguese HD and normal allele cohorts (24). In addition, a distinctively unstable C haplotype of African origin, highly associated with juvenile HD onset, has been recently described (22,23). It is, therefore, plausible that *HTT* haplotypes might display different susceptibility to germline expansion, ranging from a low instability for haplotype A1, to a major predisposing influence to larger expansion among A2 and some chromosomes of haplogroup C. It is also possible, however, that the high prevalence of A2 alleles among HD chromosomes in our population might have been the consequence of a combination of factors; that is, a high allele instability (circumstance shared with haplotype C), plus the presence of a high-normal and IA reservoir (as observed among A1 chromosomes). Unfortunately, we are not aware of any other *HTT* haplotype distribution studies across different CAG repeat ranges in these or other populations to compare with our results, thus, conclusions need to await for additional studies in other populations.

The influence of *cis*- and *trans*-acting genetic modifiers on HD clinical outcomes has been an area of research in the last years. Recent studies have analyzed the sequence immediately downstream of the CAG tract (11–13,25), identifying variants with the greatest impact of all previously identified modifiers of age at onset. We investigated exon one sequence, seeking variants that might act as *cis*-regulatory elements, contributing to the different germline instability patterns observed in the Spanish haplotypes. Consistent with other studies (11), the frequency of atypical structures in alleles of < 27 CAGs was higher (7%) than in intermediate and expanded alleles (5%). Interestingly, however, allele lengths seem to be related to the presence of particular variants. Although alleles of < 27 CAGs mainly carry two common atypical structures, those of ≥27 CAGs present higher heterogeneity. These results, which are in line with previous reports (11), point at a unique genetic backbone of some larger alleles that might make them more prone to the CAG tract expansion. Alternatively, it is possible that atypical allele structures arise as secondary mutational products of already expanded alleles.

Deletions of CAACAG and CAACAGCCGCCA, have been described as major modifiers of age at onset (11–13), and high intergenerational instability (12,13,26–28). These variants, which comprised 1.2% of IAs and 2% of HD chromosomes in our cohort, were similarly associated with early onset of symptoms and large CAG transmissions (+9, +6 CAGs). Interestingly, these structures were only found in haplogroup C (3/4) and haplotype A2 (1/4), the haplotypes with the highest level of CAG intergenerational instability in our cohort. In addition, they comprised 10% and 9% of haplogroup C alleles among the expanded and intermediate CAG ranges, respectively, and they were associated to families with unstable transmissions. Representation of these deletions among A2 chromosomes (1/63 ≥ 27 CAGs), however, was much less notable, and they could not explain CAG instability of families with large expansions. Consequently, the presence of other factors and/or *cis*-regulatory elements are required to explain differential CAG tract instability of HD chromosomes.

As these non-canonical variants account for a very small proportion of HD and IA chromosomes, the sample size is a limitation of our study. Nonetheless, the few studies that have shown frequencies of some of these variants in different populations resemble those observed among our Spanish cohort (11). These studies are still scarce in number and mostly conducted in Caucasians; therefore, it would be interesting to extend *HTT* sequencing analysis to other populations, such as African or Asian, which would potentially widen the current knowledge of different haplotypes and *cis*-elements associated with CAG repeat instability.

In conclusion, we present an extended haplotype makeup analysis of the *HTT* gene across the normal, intermediate and expanded CAG alleles, in the Spanish population, along with an evaluation of potential haplotype-dependent germline instability factors. *HTT* Spanish haplotypes resemble those observed in other southern European populations (and potentially in the Middle East) where haplotype A2 is more frequent than A1, in contrast with what is observed in the north of Europe. Moreover, the presence of Spanish HD haplotypes C1 and C, very infrequent in north-western European ancestry HD populations but predominant in HD chromosomes of Asia and Africa, unravel a unique *HTT* gene variability in Spain, likely the result of gene flow from the north of Africa to the Iberian peninsula (29). This genetic diversity has allowed observing expansions in different haplotypes within the same population. *HTT* alleles appear to exhibit different germline expansion dynamics, with some HD chromosomes arising from a pool of IAs, while others, of a more unstable nature, may be more prone to larger expansions occurring under the enhancing effect of certain, and rare, *cis*-elements.

## Materials and Methods

### Subjects. Study populations

**Figure 5:**
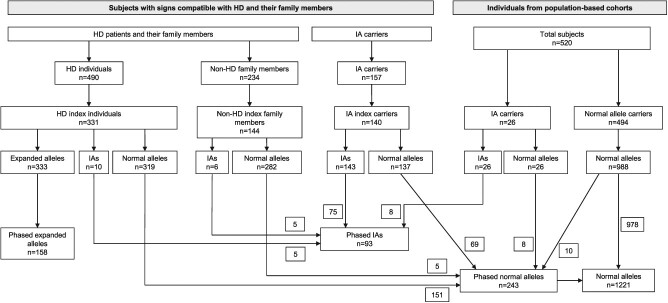
Flow diagram depicting the study populations, which include: (i) HD subjects (*n* = 490) and their family members (*n* = 234), (ii) intermediate allele (IA) carriers (*n* = 157) and (iii) individuals from population-based cohorts (*n* = 520). They comprise 991 non-related individuals: 331 HD, 140 IA carriers and 520 controls. Of them, 158 expanded, 93 intermediate and 243 normal chromosomes were CAG-phased for the haplotype study.

Study subjects were retrospectively identified at 11 reference HD centres in Spain: Hospital Clínic i Provincial de Barcelona, Fundación Jiménez Díaz, Hospital Universitario de Navarra, Hospital Universitario Central de Asturias, Hospital Universitari Son Espases, Hospital de la Santa Creu i Sant Pau, Hospital Universitario Cruces, Hospital Universitario Donostia, Hospital Universitario Ramón y Cajal, Hospital Clínico San Carlos, and Hospital Universitario y Politécnico de La Fe. The study population included subjects with signs compatible with HD and their family members referred for genetic testing and counselling, and individuals (*n* = 520) from the Spanish general population. The former group was classified into: (i) HD cases (*n* = 490) and their family members (*n* = 234), and (ii) IA carriers (*n* = 157). A detailed diagram depicting the study population is shown in Figure 5. For cases identified by genetic centres, data on family history, age at onset of neurological symptoms and age at sampling were recorded. In all families, one index member was designated (first subject identified in the family). For the general population study, we used anonymized banked DNA sample cohorts from four different Spanish regions (Asturias, Cataluña, Navarra and Basque Country), and individual age at sampling and sex information, when available.

This study was approved by the Ethics Committee of Clinical Research of Navarra (Comité de Ética de la Investigación con Medicamentos), with project number PI_2015/78.

### CAG repeat sizing

All DNA samples included in the study were re-analysed using an identical CAG repeat sizing assay, performed at the Department of Medical Genetics in Pamplona. CAG repeat length was determined using previously established PCR amplification assays (30,31) with fluorescently-labelled primers flanking the CAG repeat sequence. The locus-specific primers used were: F 5′-ATGGCGACCCTGGAAAAGCTGATGAA-3′ and R 5′-GGCGGTGGCGGCTGTTGCTGC-3′ (6-FAM fluorescent label). PCR products were separated by capillary electrophoresis with 3500 Genetic Analyzer (Applied Biosystems) and the fragment size was determined with GeneMapper Software 5.

Alleles were classified as normal (<27 CAG), intermediate (27–35 CAG), or HD (>35 CAG). Within normal alleles, those with 20–26 CAG were considered high-normal alleles. HD-associated CAG repeat alleles were further classified as reduced penetrance (36–39 CAG) or full-penetrance (>39 CAG).

### SNP selection and haplotype determination

**Figure 6:**
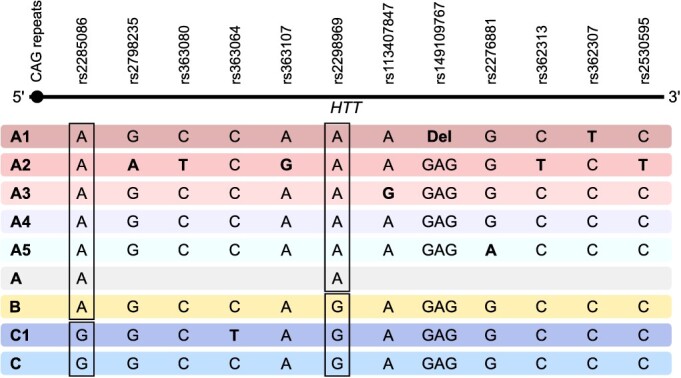
Schematic map of *HTT* gene displaying the genotypes of the selected SNPs that define A/B/C haplogroups (boxes) and the different haplotypes.

Thirty-four SNPs (Supplementary Material, Table S1) were genotyped in 490 HD patients, 183 IA carriers, and 728 normal allele carriers, using the Agena Multiplex (CEGEN, Santiago de Compostela, Spain) and LGC Kasp (LGC, Hoddesdon, UK) technologies. Of them, we selected 12 haplotype-representative SNPs (21), which, after haplotype construction with PHASE 2.0 (32), characterized over 98% of HD and general population chromosomes. Haplogroups A, B and C were defined with rs2285086 and rs2298969, haplotype A1 was defined with rs149109767 and rs362307, A2 with rs2798235, rs363080, rs363107, rs362313, rs2530595, A3 with rs113407847, A4 or A5 with rs2276881 and C1 with rs363064. Haplotype genotypes are detailed in Figure 6.

Phasing CAG repeats with *HTT* haplotypes was conducted using data on familiar segregation, when available. Otherwise, phasing was determined based on the CCG repeat size association with specific haplotypes (9,21) and/or with specific sequences downstream of the CAG repeats (13,33). Haplotype frequencies were calculated using an index individual per family.

### *HTT* exon one sequencing and genotyping

MiSeq sequencing libraries of the *HTT* exon one were generated using MiSeq system-compatible primers and sequencing was performed according to the protocol described by Ciosi *et al.* (34). For the sequencing library preparation, the *HTT* exon one repeat region was amplified from 20 ng of DNA extracted from the peripheral blood of 401 individuals in the study population carrying, at least, one allele of ≥27 CAGs.

The output was analyzed with ScaleHD (v0.251), as previously described (11). Genotyping was determined and alleles were classified as typical or atypical. The variant annotation was based on the National Center for Biotechnology Information nomenclature of the *HTT* locus reference sequence NM_001388492. Typical alleles, or alleles with the canonical sequence, were defined as those where the polymorphic CAG repeat sequence was followed immediately downstream by CAACAGCCGCCA, which was then followed by a polymorphic CCG repeat sequence and then followed by CCTCCT. Thus, the nomenclature of a typical allele structure was defined as (CAG)*_x_*(CAACAG)_1_(CCGCCA)_1_(CCG)*_y_*(CCT)_2_.

### Families with large intergenerational CAG tract length changes

HD family pedigrees were investigated to search for unstable CAG transmissions. Families with an intergenerational variation of >5 CAGs were selected and further information was sought to extend familiar, molecular and clinical data.

### Statistical analysis

Statistical analysis was performed using IBM SPSS Statistics for Windows, Version 20.0. Results from CAG tract sizes, allele structures and haplotypes were summarized using descriptive statistics, such as percentages, mean and SD. CAG distribution differences between independent groups were analyzed with *t*-test for independent samples. The variation in CAG distributions among different haplotypes was determined by ANOVA test. Linear regression, adjusted by the number of CAGs, was performed to examine the effect of the different exon 1 variants on age at onset, and 95% confidence intervals (95% CI) were estimated. The association between two categorical variables was determined with Fisher’s exact test. In order to assess the accuracy and robustness of the estimators derived from the original dataset, *P*-values and 95% CI were re-calculated by bootstrapping, i.e. by random sampling with replacement from the original data, using 1000 replications and the percentile method. All tests were two-sided and a *P*-value < 0.05 was considered statistically significant.

## Supplementary Material

Supplemental_data_ddac224
