# Supplementary material for: Spanish HTT gene study reveals haplotype and allelic diversity with possible implications for germline expansion dynamics in Huntington disease
Source: Hum Mol Genet. 2022 Sep 20;32(6):897–906. doi: 10.1093/hmg/ddac224 (PMC9990985; doi:10.1093/hmg/ddac224)
Supplement: Supplemental_data_ddac224 [file supplemental_data_ddac224.docx]

**Supplementary Table 1** Summary of statistical results comparing the p-values and 95% CI derived from: a) the original dataset, and b) the bootstrap resampling.

|  | Statistical test | Original dataset | | Bootstrapped (B=1000) | |
| --- | --- | --- | --- | --- | --- |
|  |  | p-value | b (95% CI) | p-value | b (95% CI) |
| 1. CAG length distribution (Table 1) | | | | | |
| Population-based cohorts (n=1040) vs small allele of IA carriers (n=140) | Independent samples t-test | 0.973 | 0.010  (-0.570, 0.590) | 0.964 | 0.010  (-0.574, 0.600) |
| Population-based cohorts (n=1040) vs small allele of HD subjects (n=331) |  | 0.079 | b=0.376  (-0.043, 0.796) | 0.100 | b=0.376  (-0.101, 0.820) |
| 2. CAG length distribution by haplotype (A1, A2, A3, A4, A5, B, C1, C) (Table 2) | | | | | |
| Small alleles of IA carriers and HD subjects (n=252) | ANOVA | <0.001 | - | <0.001 | - |
| Expanded alleles (n=158) |  | 0.067 | - | 0.071 | - |
| 3. Onset of symptoms - linear regression | | | | | |
| (CAA)_1_ (n=62) vs (CAA)_0_ (n=3) | Linear regression adjusted by No.of (CAG), with No. of (CAA) as a factor | 0.011 | -15.266  (-26.886, -3.646) | 0.008 | -15.266  (-24.510, -0.758) |
| (CAA)_1_ (n=62) vs (CAA)_2_ (n=3) |  | 0.522 | 3.686  (-7.739, 15,110) | 0.129 | 3.686  (-1.066, 10.720) |
| 4. % CAACAG deletion | | | | | |
| CAACAG deletion in unstable HD families (2/14), vs non-unstable HD families (1/93) | Fisher’s exact test | 0.044 | - | 0.067 | - |

**Supplementary Table 2** List of genotyped SNPs.

| **RS Number** | **Position (GRCh37)** |
| --- | --- |
| rs2857845 | chr4:3028113 |
| rs2471347 | chr4:3044435 |
| rs13141939 | chr4:3061282 |
| rs2798296 | chr4:3062165 |
| rs3856973 | chr4:3080173 |
| rs2285086 | chr4:3089259 |
| rs10015979 | chr4:3109442 |
| rs2798235 | chr4:3114832 |
| rs2071655 | chr4:3118116 |
| rs363082 | chr4:3132713 |
| rs363081 | chr4:3133627 |
| rs363080 | chr4:3133911 |
| rs363064 | chr4:3141410 |
| rs363107 | chr4:3144441 |
| rs6855981 | chr4:3148276 |
| rs35892913 | chr4:3148570 |
| rs11731237 | chr4:3151813 |
| rs363096 | chr4:3180021 |
| rs2298969 | chr4:3186244 |
| rs363092 | chr4:3196029 |
| rs113407847 | chr4:3211842 |
| rs362331 | chr4:3215835 |
| rs916171 | chr4:3216815 |
| rs362325 | chr4:3219326 |
| rs82333 | chr4:3225389 |
| rs110501 | chr4:3225478 |
| rs149109767 | chr4:3230411 |
| rs2276881 | chr4:3231661 |
| rs362272 | chr4:3234980 |
| rs362313 | chr4:3235589 |
| rs362307 | chr4:3241845 |
| rs2530595 | chr4:3245057 |
| rs3129322 | chr4:3252852 |
| rs3095073 | chr4:3263138 |
